# Digital gene expression analysis of male and female bud transition in *Metasequoia* reveals high activity of MADS-box transcription factors and hormone-mediated sugar pathways

**DOI:** 10.3389/fpls.2015.00467

**Published:** 2015-06-24

**Authors:** Ying Zhao, Haiying Liang, Lan Li, Sha Tang, Xiao Han, Congpeng Wang, Xinli Xia, Weilun Yin

**Affiliations:** ^1^National Engineering Laboratory for Tree Breeding, College of Biological Sciences and Biotechnology, Beijing Forestry UniversityBeijing, China; ^2^Department of Genetics and Biochemistry, Clemson UniversityClemson, SC, USA

**Keywords:** DGE, *Metasequoia*, male and female bud, MADS-box, sugar, hormone

## Abstract

*Metasequoia glyptostroboides* is a famous redwood tree of ecological and economic importance, and requires more than 20 years of juvenile-to-adult transition before producing female and male cones. Previously, we induced reproductive buds using a hormone solution in juvenile *Metasequoia* trees as young as 5-to-7 years old. In the current study, hormone-treated shoots found in female and male buds were used to identify candidate genes involved in reproductive bud transition in *Metasequoia*. Samples from hormone-treated cone reproductive shoots and naturally occurring non-cone setting shoots were analyzed using 24 digital gene expression (DGE) tag profiles using Illumina, generating a total of 69,520 putative transcripts. Next, 32 differentially and specifically expressed transcripts were determined using quantitative real-time polymerase chain reaction, including the upregulation of MADS-box transcription factors involved in male bud transition and flowering time control proteins involved in female bud transition. These differentially expressed transcripts were associated with 243 KEGG pathways. Among the significantly changed pathways, sugar pathways were mediated by hormone signals during the vegetative-to-reproductive phase transition, including glycolysis/gluconeogenesis and sucrose and starch metabolism pathways. Key enzymes were identified in these pathways, including alcohol dehydrogenase (*NAD*) and glutathione dehydrogenase for the glycolysis/gluconeogenesis pathway, and glucanphosphorylase for sucrose and starch metabolism pathways. Our results increase our understanding of the reproductive bud transition in gymnosperms. In addition, these studies on hormone-mediated sugar pathways increase our understanding of the relationship between sugar and hormone signaling during female and male bud initiation in *Metasequoia*.

## Introduction

*Metasequoia glyptostroboides* Hu et Cheng, a famous living fossil (also known as the dawn redwood) and the sole extant species of *Metasequoia*, which has been merged with Cupressaceae as the subfamily Sequoia (Brunsfeld et al., 1994), is found in the Hubei and Hunan provinces in south-central China, and has been widely distributed in the Northern Hemisphere (Nelson, 1998; Kunzmann and Mai, 2011).

Although of great ecologic and economic importance, like most gymnosperm trees, *Metasequoia* has a long juvenile phase (20–30 years) before female bud appearance, making their seeds scarce. It is a monoecious species with male cones and female cones on different twigs of adult trees. In the past few decades, there have been studies on morphology and anatomy (Sterling, 1949; Dörken, 2012); however, the majority of these have focused on female cones. Few reports have examined male cones (e.g., Jin et al., 2012) because few trees produce them. In addition, despite its large numbers and worldwide distribution, most adult trees have low seed viability as a result of inbreeding depression (Li et al., 2005). Given the fact that male bud initiation is functionally adapted for successful pollination, exploring the gene expression involved in male reproductive development is important for understanding the molecular regulation of flowering and cone development, and for determining how and why there are few male cones on adult trees. At this time, few studies are available on *M. glyptostroboides* due to limited genomic resources. The only report on *Metasequoia* genomic resources is on an expressed sequence tag (EST) dataset built from both vegetative buds and reproductive buds during the late stages of bud development (Zhao et al., 2013). Thus, *Metasequoia* behind lagged behind other gymnosperm species such as Norway spruce, White spruce, and loblolly pine in genomic resources (Birol et al., 2013; Nystedt et al., 2013; Neale et al., 2014; Zimin et al., 2014).

Physiological and genetic studies have demonstrated that vegetative-to-reproductive transitions are not only regulated by environmental signals such as day length, light intensity, and ambient temperature (Trevaskis et al., 2006; Winfield et al., 2009), but are also regulated by endogenous signals such as plant hormones and age (Wang et al., 2011; Poethig, 2013). We previously found that spraying hormones (GA, IAA, ABA) on 5 year old trees could induce female and male buds and shorten this phase (Zhao et al., 2013). Recent studies have revealed the molecular mechanism of the phase between vegetative and reproductive transitions in annual species of *Arabidopsis* and maize, as well as some perennial wood species. In *Arabidopsis*, GA3 (Gibberellin) treatment can activate SUPPRESSOR OF CONSTANS1 (SOC1), which belongs to the MADS-box family of transcription factors, to induce flowering (Moon et al., 2003). Thus, MADS-box transcription factors are essential for the flowering program. Previous studies showed that many interactions between MADS-domain proteins are conserved among *Arabidopsis thaliana*, rice (*Oryza sativa*), petunia (*Petunia hybrida*), and *Antirrhinum majus* (Ferrario et al., 2004). Based on advances in our understanding of the genetic network controlling flowering in *Arabidopsis*, various biotechnological approaches have been used to shorten the juvenile phase in angiosperm trees (Bohlenius et al., 2006). However, recent studies have suggested that regulation of the vegetative-to-reproductive switch may differ in gymnosperms compared to angiosperms (Klintenas et al., 2012). Studies on the MADS-box gene family in gymnosperms suggest that some members in this family play an important role in shortening the juvenile phase and specifying reproductive identity in the male and female buds (Carlsbecker et al., 2004).

Sugar can provide energy for the plant, and act as a signal in coordination with hormone signaling pathways to control various plant physiological processes (Rolland et al., 2006). However, no sugar analysis was carried out in Metasequoia. Distinct glycolysis/gluconeogenesis, starch and sucrose metabolism pathways, and fructose signaling pathways have been examined (Cho and Yoo, 2011; Li et al., 2011). These pathways are regulated by various sucrose enzymes, including trehalose-6-phospath (T6p), UDPGluc, and glucose 6-phosphate (G6p) (Lunn et al., 2006; Huang et al., 2012). Sugar signaling plays an important role in controlling flowering time, and affects juvenile-to-adult phase transition in plants (Yu et al., 2013). Recently, the relationship between sugar metabolism/signaling and floral transition has been explored (Turnbull, 2011; Moghaddam and Van-den-Ende, 2013). However, the molecular mechanism controlling the transition to flowering through the hormone-mediated sugar pathway remains unclear.

In the present study, we performed digital gene expression (DGE) tag profiling using the Illumina next-generation sequencing (NGS) platform, taking advantage of its sensitivity for transcript analysis and high efficiency to sequence expressed genes compared to cDNA microarrays (Wang et al., 2009; Matsumura et al., 2010; Robinson et al., 2010). We mapped the Illumina reads to the previous 454 transcriptomic data, and obtained expression data for 69,520 putative unique transcripts (PUTs) and 243 associated Kyoto Encyclopedia of Genes and Genomes (KEGG) pathways of *Metasequoia*. Furthermore, we compared the gene expression profiles of vegetative and reproductive buds (male and female) in different stages of bud formation, and identified numerous differentially and specifically expressed transcripts at different developmental stages of bud formation. Our study provides new information on the transcriptome analysis of *Metasequoia* buds, demonstrating the use of DGEs to examine transcript abundance of male and female buds in the early stage of bud initiation, and providing a valuable resource for studying gene function in gymnosperms.

## Materials and methods

### Hormone treatment and sample collection

Hormone (indole butyric acid, zeatin ribonucleoside, 0.5% Tween-20) treatments were conducted on 38 years old *M*. *glyptostroboides* trees located on the campus of Beijing Forestry University (latitude 40.0 N, longitude 116.2 E, 57 m above sea level) using the spray method, as previously described (Zhao et al., 2013). The treatments were divided into two groups in each tree, with three branches for each group. For group one, the hormone treatments were conducted at 5:00 p.m. on May 15 and May 25 in 2013. For group two, hormone treatments were conducted also at 5:00 p.m. on June 10, June 25, and July 10 in 2013. The treated shoots (TR) were sprayed until liquid drops started to drip down from the leaves, as previously described (Zhao et al., 2013). Untreated shoots (CK) in a similar location in each tree were used as the control.

Buds with the current year's stems (approximately 1 mm in length on TR and CK from the same tree) for three phenotypes were collected during bud differentiation when hormone treatment was efficacious for male bud induction on May 26 (samples TR1-1, TR1-2, TR1-3, CK1-1, CK1-2, CK1-3) and on June 11 (samples TR2-1, TR2-2, TR2-3, CK2-1, CK2-2, CK2-3), and was effective for female bud initiation on June 26 (samples TR3-1, TR3-1, TR3-3, CK3-1, CK3-2, CK3-3) and on July 11 (samples TR4-1, TR4-2, TR4-3, CK4-1, CK4-2, CK4-3). All of the samples were harvested from three individual trees (biological repeats) between 5:00 p.m. and 7:00 p.m., and were immediately frozen in liquid nitrogen. Samples were collected from all three trees for each time point, and were stored at −80°C for RNA extraction.

For the two group, some treated twigs remain for male bud and female percent calculation in winter and next spring, when it is easy for reproductive bud morphologically distinguishable with bare eyes. The estimate was done by the following formula: the percent of male (female) bud = the total number of male (female) buds/ the number of vegetative and male (female) buds.

### RNA extraction and sequencing

Total RNA from all the samples was extracted separately using Trizol reagent (Invitrogen, CA, USA) according to the manufacturer's instructions. Total RNA quantity and purity were examined by Bioanalyzer (Agilent). Approximately 10 μg total RNA representing a specific type was subjected to mRNA isolation with poly-T oligo-attached magnetic beads (Invitrogen). Following purification, the mRNA was fragmented into small pieces using divalent cations under elevated temperatures. The cleaved RNA fragments were reverse-transcribed to create the final cDNA library in accordance with the protocol for the mRNA-Seq sample preparation kit (Illumina, San Diego, USA). 24 DGE libraries were constructed with Illumina's DGE Tag Profiling Kit according to the manufacturer's protocol (Version 2.1B). The eight (average of the three replicates) cDNA libraries in our study represented vegetative bud initiation (CK1 and CK2), male reproductive buds in early development (TR1 and TR2), vegetative buds in early development (CK3 and CK4), and female reproductive buds in early development (TR3 and TR4). Then, the 24 tag libraries underwent Illumina proprietary sequencing for cluster generation through *in situ* amplification. Single-end sequencing (36 bp) was performed on an Illumina Hiseq2500 at the LC-BIO (Hangzhou, China). Raw sequence data were available in GenBank Short Read Archive (Accession SRP051608).

### DGE-tag profiling

The generated image files were processed to produce digital-quality sequence data. The raw data were filtered to obtain clean reads. The types of clean tags were represented as distinct clean tags. Subsequently, we classified the clean tags and distinct clean tags according to their copy number in the library, calculated their percentage in total clean and distinct tags, and analyzed library saturation. For annotation, all of the clean tags were mapped to unigenes of the Metasequoia database that we previously generated (http://www.genome.clemson.edu/node/273) with Bowtie2 (Langmead and Salzberg, 2012), because there is no *Metasequoia* genome sequence available.

### Assembly of different cDNA libraries and de novo transcript

All the mapped clean tags were assembled again according to the 96,565 PUTs. 454 original data without adaptors (Zhao et al., 2013) were *De novo* assembled again with Trinity (Haas et al., 2013).

### Identification of DE genes

The expression level of each gene was normalized to RPKM based on the number of clean tags. Genes were deemed significantly DE with a *P* < 0.05 (FDR < 0.1), and a relative change threshold of two-fold in the sequence counts across libraries. To obtain reliable and precise expression levels, the average of three replicates of RPKMs was used to determine the final fold change. All DE genes were divided into 16 comparisons (CK1 vs. CK2, CK1 vs. CK3, CK1 vs. CK4, CK2 vs. CK3, CK2 vs. CK4, CK3 vs. CK4; TR1 vs. TR2, TR1 vs. TR3, TR1 vs. TR4, TR2 vs. TR3, TR3 vs. TR4; CK1 vs. TR1, CK2 vs. TR2, CK3 vs. TR3, CK4 vs. TR4). All of the downregulated or upregulated genes in the following description were regulated in the second comparison component. Functional classification of DE genes was performed according to the functional categories of Gene Ontology (GO).

### Biological pathway analysis

GO-defined pathways using the global test (available from Bioconductor: www.bioconductor.org) (Goeman et al., 2004) were significantly deregulated for comparisons. After summarization of the tags for each Entrez Gene entry, a global test was performed on the scaled and square root transformed data. *P*-values were calculated using the asymptotic method. Additional filtering of pathways was performed using the median of z-scores for each gene in the pathway to identify significant pathways.

### Phylogenetic analysis

Annotated MADS box genes with high similarity to *Metasequoia* PUTs putatively for MADS-protain from selected angiosperms and gymnosperms were retrieved from GenBank using BLAST searches. We obtained amino acid sequences and aligned them using the Clustal W program in Mega 5.0. A neighbor-joining (NJ) tree was obtained and local bootstrap probability of each branch was estimated with Mega 5.0 (Tamura et al., 2011).

### Real-time PCR analysis

To verify the DGE results, a total of 32 *Metasequoia* PUTs were selected for quantitative RT-PCR (qRT-PCR) analysis using the same plant materials used for sequencing (three distinct biological samples). These transcripts were selected because of their high abundance in the cDNA libraries according to RNA sequencing. cDNA synthesis was performed using the first Strand cDNA Synthesis Kit (TaKaRa) with Oligo (dT) primers. The qRT–PCR experiments were performed on a StepOnePlus (Applied Biosystems) with SYBR Green supermix (Applied Biosystems). Three replicates were included for each sample. The reactions contained 45 cycles of 15 s at 95°C and 30 s at 60°C, followed by the thermal dissociation protocol for SYBR green detection. PCR Miner was used to analyze the raw data (Zhao and Fernald, 2005), and to calculate the cycle threshold (*Ct*-value) and PCR efficiency. The 2-ΔΔCT method (Schmittgen and Livak, 2008) was used to calculate the relative expression level of the gene of interest. 18S rRNA was used as the internal control. The primer sequences used are shown in Supplementary data 1.

### Measurements of non-structural carbohydrates

A total of 1.5 g of hormone-treated and untreated Metasequoia stems with buds (1 mm stem in length) were weighed and ground with mortar and pestle, separately. Five milliliters of 80% methanol (v/v) (0.1 M with pH 7) with 1.5 g samples was added into glass tube, incubated at 70°C for 15 min, and then centrifuged at 10,000 rpm for 10 min. This extraction was repeated twice. 0.5 ml of an internal standard (methyl-^a^-D-glucopyranoside, 10 g/250 ml) was added to the supernatant, and then centrifuged at 10,000 rpm for 5 min. 80% methanol was then added until a volume of 25 ml. About 5 ml supernatant was concentrated to an anhydrous state after atmospheric distillation at 45°C for about 20 min. Then 1 ml derivatization reagent was added for solving for 15 min, and 0.5 ml was used for GC analysis (Bureau et al., 2013).

## Results

### Hormone-treated shoots show male buds and female cones

**Figure 1:**
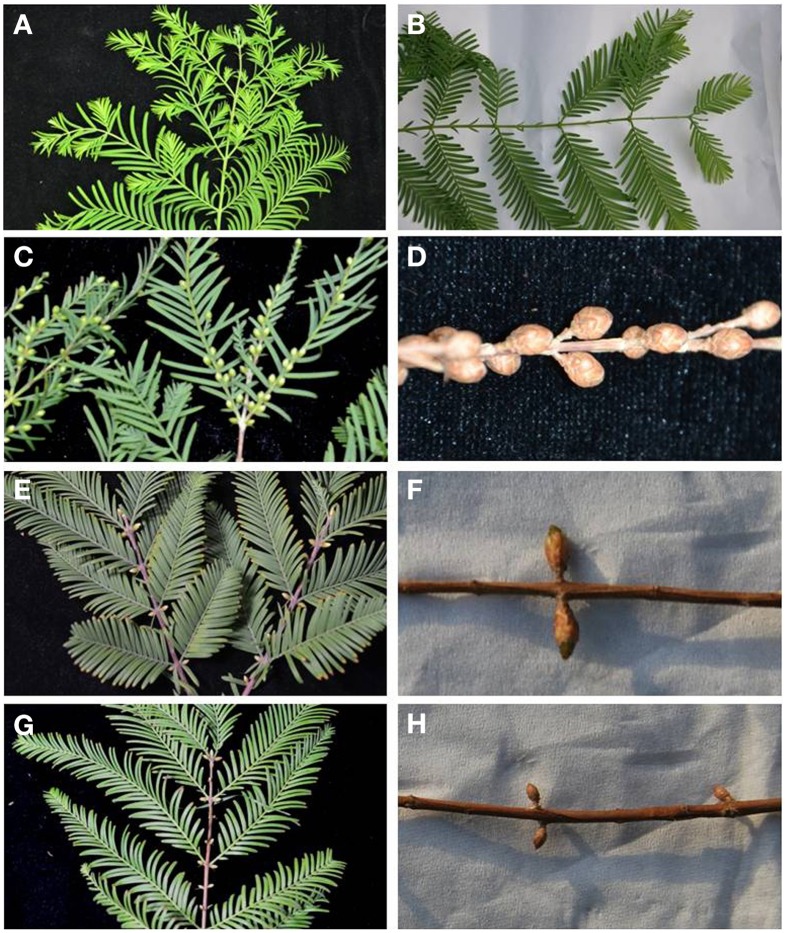
**Male buds and female cones on hormone-treated shoots. (A)** Shoots before hormone treatment collected on May 24th, 2013. **(B)** Shoots before hormone treatment collected on June 11th, 2013. **(C)** Male buds on treated shoots collected on Sept 15th, 2013. **(D)** Male cones on treated shoots collected on Feb 5th, 2014. **(E)** Female buds on treated shoots collected on Oct16th, 2013. **(F)** Female buds on treated shoots collected on Mar 5th, 2014. (**G**) Untreated shoots (control) with vegetative buds collected on Oct 16th, 2013. **(H)** Untreated shoot (control) with vegetative buds collected on Mar 5th, 2014.

According to our observation, in 2014, approximately 70% of female cones (Figures 1E,F) were found in June and July hormone treated shoots and the other half were vegetative buds. The shoots sprayed with hormone in May produced about 50% of male buds (Figures 1C,D), and the others were vegetative buds (Figures 1G,H). None of reproductive buds were observed on the control branches and on the shoots before hormone treatment (Figures 1A,B). The rectangle and elliptical female buds are 2–4 mm longer than the obovate male buds. The male cones were initiated in the axils of opposite leaves, with two male cones per leaf pair. Longitudinal sections of male buds shows pollen while female buds meristem were shown on the micrographs of female buds (Supplementary Figure S1).

### Sequence analysis and assembly of different cDNA libraries

**Figure 2:**
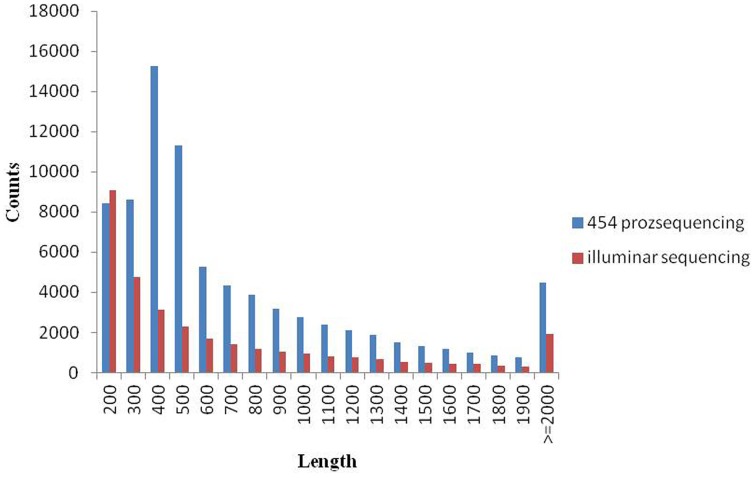
**The size (bp) distribution of PUTs**.

Since there is no *Metasequoia* genome sequence available, the transcripts from different samples were reconstructed *ab initio* using a comprehensive set of 97,565 *Metasequoia* PUTs as a reference (Zhao et al., 2013). Based on 97,565 PUTs, we detected expression data for 69,520 genes, covering 71.3% of previous databases. After assembly, 32,186 PUTs at least 200 bp with an average of 740 bp (N50 = 1132) were generated. The original 454 data (Zhao et al., 2013) without adaptors were *de novo* assembled again. As a result, we generated 80,514 PUTs at least 200 bp in length with an average length of 807 bp (N50 = 1046). The size (bp) distribution of PUTs is represented in Figure 2. After assembly, 31,405 PUTs were mapped to the reference EST dataset of *Metasequoia*. The aligned sequences could be found in Supplementary data 2.

Our sequencing on an Illumina Genome Analyzer II generated a range of 25.7–50.2 million clean reads per library. When mapped to the 97,565 *Metasequoia* PUTs (http://www.genome.clemson.edu/node/273, Zhao et al., 2013), the number of alignable reads ranged from 14.6 million (CK1 on May 26, 56.7%) to 38.9 million (TR2 on June, 77.4%) based on clean reads. Among the alignable reads, 7.8–15.9 million were uniquely aligned reads, with CK1 making up the highest percentage (53.7%) (Table 1).

**Table 1:** **Summary of RNA-seq and mapping results of** ***Metasequoia*** **reads**.

**Date of collection**	**Treatment^a^**	**No. of clean reads**	**Mapped to transcriptome of *Metasequoia*^b^**	
			**No. of alignable reads^c^**	**No. of uniquely aligned reads^d^**
May 26	TR1	25,976,323	18,258,549 (70.3%)	8,602,093 (47.1%)
	CK1	25,719,336	14,585,788 (56.7%)	7,830,101 (53.7%)
June 11	TR2	50,225,947	38,878,052 (77.4%)	9,898,177 (25.5%)
	CK2	41,211,897	31,883,984 (77.3%)	14,753,296 (46.3%)
June 26	TR3	37,263,380	22,416,081 (60.2%)	11,252,881 (50.2%)
	CK3	27,915,451	21,429,218 (76.8%)	7,918,341 (37.0%)
July 11	TR4	48,134,744	34,868,085 (72.4%)	15,892,835 (45.6%)
	CK4	36,537,778	24,873,981 (68.1%)	12,285,825 (49.4%)

a *Each TR or CK treatment includes three biological replicates; TR stands for hormone treatment and CK stands for the non-treatment control*.

b *(Zhao et al., 2013)*.

c *The numbers in parentheses are the percentage of reads based on the number of total clean reads*.

d *The numbers in parentheses are the percentage of reads based on the number of alignable reads*.

### Go annotation and DE genes

**Figure 3:**
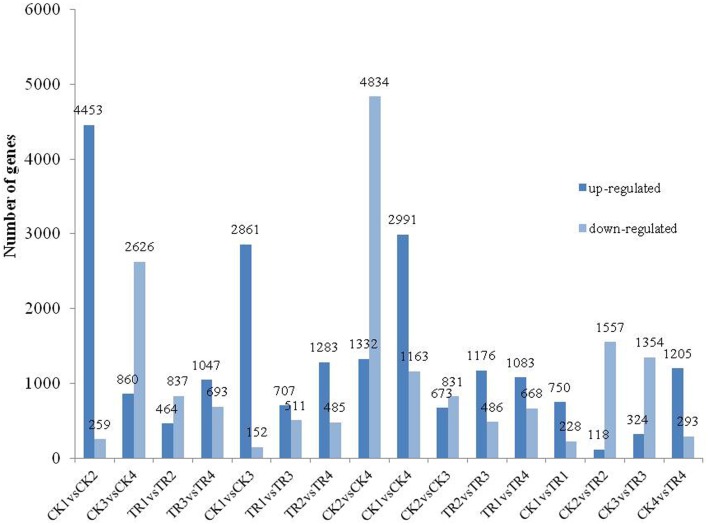
**Differentially expressed genes in different comparisons**.

**Figure 4:**
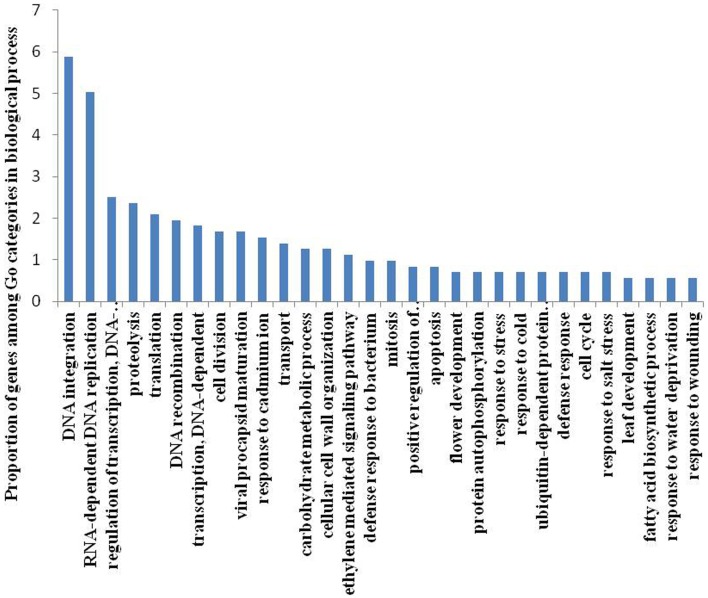
**Gene classification based on gene ontology (GO) for differentially expressed genes in** ***Metasequoia*** **in CK4 vs. TR4 comparison in biological processes**. The frequency of GO terms was analyzed using GO slim assignment. The x-axis and y-axis indicate the names of clusters and the percentage of each cluster, respectively.

To identify DE genes between reproductive and vegetative buds, we analyzed changes in expression levels among 69,520 transcripts. Statistical analyses with an adjusted *P*-value cutoff of 0.05 revealed PUTs with differential expression in all 16 comparisons (CK vs. CK, CK vs. TR, and TR vs. TR) (Figure 3). Comparisons with vegetative buds showed the most DE PUTs (6166). For CK and TR comparisons group, the highest number of DE PUTs was found in CK3 vs. TR3 (June 26) (1678), and CK1 vs. TR1 (May 26) showed the least (978) DE PUTs. For all of the comparisons, the most enriched categories in Biological Process, Cellular Component, and Molecular Functions were DNA integration, Nucleus, and ATP binding, respectively. Genes with expression responses covered a variety of regulatory and metabolic processes. All DE genes in each comparison were classified into several categories based on GO terms (http://www.geneontology.org/). In CK4 vs. TR4 comparison, genes related to DNA integration (5.87%) and RNA-dependent DNA replication (5.03%) were most abundant in DE genes. It should be noted that approximately 0.7% of DE genes were categorized as flower development (Figure 4).

### DGE analysis shows upregulation of MADS-box genes and downregulation of a flowering time control protein (FPA) involved in early male bud initiation

**Figure 5:**
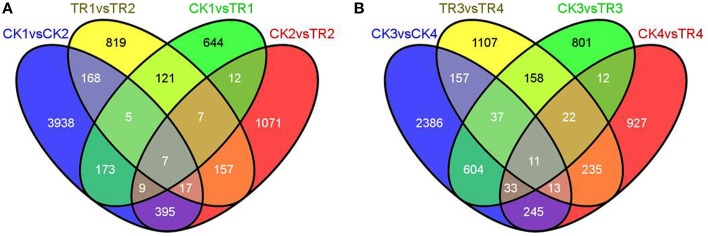
**Venn diagrams with numbers of shared and unique differentially expressed genes. (A)** DE genes between CK1 vs. TR1, CK2 vs. TR2, CK1 vs. CK2, and TR1 vs. TR2. **(B)** DE genes between CK3 vs. TR3, CK4 vs. TR4, CK3 vs. CK4, and TR3 vs. TR4.

By comparing transcript abundance in samples from shoots undergoing male bud initiation with samples from CK, 978 (750 upregulated and 228 downregulated, CK1 vs. TR1) and 1675 (118 upregulated and 1557 downregulated, CK2 vs. TR2) significantly DE transcripts were identified in late May and early June, respectively (Figure 5A). When combining the two data sets, 35 transcripts were significantly upregulated or downregulated in TR for male bud initiation. Seven of these genes were also DE (CK1 vs. CK2) in control and (TR1 vs. TR2) treated plants. The gene ID and annotation of the 35 DE genes are listed in Supplementary data 3. Among the 978 DE genes in CK1 vs. TR1 comparison, we focused on two putative transcripts; isotig33092, and isotig32790, both of which were upregulated in TR. By examining these genes, we found a decrease in the transcript isotig33092 putatively encoding Agamous-like MADS-box protein (AGL21) in CK4 when compared to CK2. Another transcript isotig32790 putatively coding for MADS-box transcription factor 50 showed downregulation of TR3 compared to TR1, downregulation of TR4 compared to TR1, and downregulation of CK4 compared to CK2. Among the 1675 DE genes in CK2 and TR2, two transcripts: isotig12610 and isotig12611, both putatively encoding *FPA*, were downregulated in TR2. When compared among controls, these two transcripts were downregulated in CK4 compared to CK2, but were upregulated in CK2 compared to CK1. Based on these results, these six genes showed enhanced male bud initiation in *Metasequoia* (Table 2).

**Table 2:** **Six differentially expressed genes identified in hormone-treated shoots undergoing male bud initiation**.

**Comparisons**	***Metasequoia* PUTs ID**	**Differential expression**	**Arabidopsis or Oryza match**	**Annotation**
CK1 vs. TR1	isotig42852	Down	ELF3	Protein EARLY FLOWERING 3
	isotig34815	Down	Os08g0159800	Zinc finger CCCH domain-containing protein 56
	isotig33092	Up	AGL21	Agamous-like MADS-box protein AGL21
	isotig32790	Up	MADS50	MADS-box transcription factor 50
CK2 vs. TR2	isotig12610	Down	FPA	Flowering time control protein FPA
	isotig12611	Down	FPA	Flowering time control protein FPA

### DGE analysis indicates upregulation of zinc finger CCCH domain-containing protein family factors and FCA during the female bud initiation stage

By comparing transcript abundance in samples from shoots for female buds with samples from CK, 1678 (324 upregulated and 1354 downregulated, CK3 vs. TR3) and 1498 (1205 upregulated and 293 downregulated, CK4 vs. TR4) significantly DE transcripts were identified, respectively (Figure 5B). When combining the dataset of non-female bud formation comparisons with female bud shoots comparisons, 44 transcripts were common in CK3 vs. TR3, CK4 vs. TR4, and CK3 vs. CK4. We further characterized a transcript isotig14067, which was downregulated in TR3 compared to CK3, upregulated in TR4 compared to CK4, and downregulated in the vegetative bud formation stage (CK4 compared to CK3). This transcript putatively encoded zinc finger CCCH domain-containing protein 28, which plays a significant role in developing floral tissue. Excluding this gene, another six transcripts putatively coding for zinc finger proteins were DE in two stages for female bud formation comparisons (CK3 vs. TR3 and CK4 vs. TR4). Other than these transcripts, two similar transcripts isotig14235 and isotig14236 putative encoding *FCA* were upregulated in TR4 compared to CK4, but were downregulated in CK4 compared to CK3. Two transcripts (isotig13796 and isotig23456) putatively coding for EARLY FLOWERING 3 (*ELF3*) and *FPA* were downregulated in both comparisons (CK3 vs. TR3 and CK3 vs. CK4). A transcriptional co-repressor LEUNIG protein (*LUG*) was upregulated in TR3 compared to CK3. We believe that these 12 genes constitute a reasonable set of candidate genes involved in the early female bud initiation in *Metasequoia* (Table 3).

**Table 3:** **Twelve differentially expressed genes identified in hormone-treated shoots undergoing female bud initiation**.

**Comparisons**	***Mg*PUTs ID**	**Differential expression**	**Arabidopsis or Oryza match**	**Annotation**
CK3 vs. TR3	isotig13796	Down	ELF3	Protein EARLY FLOWERING 3
	isotig23456	Down	FPA	Flowering time control protein FPA
	isotig10554	Down	Os11g0472000	Zinc finger CCCH domain-containing protein 63
	isotig12601	Down	At2g05160	Zinc finger CCCH domain-containing protein 18
	isotig12602	Down	At2g05160	Zinc finger CCCH domain-containing protein 18
	isotig14067	Down	Os04g0438700	Zinc finger CCCH domain-containing protein 28
	isotig78340	Up	LUG	Transcriptional corepressor LEUNIG
CK4 vs. TR4	isotig14235	Up	FCA	Flowering time control protein FCA
	isotig14236	Up	FCA	Flowering time control protein FCA
	isotig08983	Up	Os07g0682400	Zinc finger CCCH domain-containing protein 53
	isotig14067	Up	Os04g0438700	Zinc finger CCCH domain-containing protein 28
	isotig23877	Up	Os05g0195200	Zinc finger CCCH domain-containing protein 35
	isotig26834	Up	Os04g0665700	Zinc finger CCCH domain-containing protein 31

### Upregulation of genes involved in the vegetative-to-reproductive phase transition

Based on GO annotation, we selected 7 DE genes with higher expression levels in the TR for male bud initiation (CK1 vs. TR1), and 10 DE genes were upregulated in TR for female bud initiation (CK3 vs. TR3 and CK4 vs. TR4). Based on our results, these genes were activated during the transition phase. Among the genes isotig14814 putatively encodes a *LRR* receptor-like protein involved in the vegetative-to-reproductive phase transition of meristem. Isotig29943 putatively codes RNA pseudourine synthase 2, which plays a role in spermatogenesis (Table 4).

**Table 4:** **Upregulated genes in hormone-treated shoots with annotations in the vegetative to reproductive phase transition and flowering development**.

**comparisons**	***Mg*PUTs ID**	**Go annotation**	**Log_2_**-**fold**	**Annotation**
CK1 vs. TR1	isotig07103	GO:0009908 (flower development), GO:0010154 (fruit development)	1.83	Myb-related protein MYBAS1
	isotig14814	GO:0010228 (vegetative to reproductive phase transition of meristem)	1.35	LRR receptor-like serine/threonine-protein kinase ERL
	isotig15725	GO:0009553 (embryo sac development)	0.53	Very-long-chain enoyl-CoA reductase
	isotig16158	GO:0048575 (short-day photoperiodism, flowering), GO:0010182 (sugar mediated signaling pathway)	0.25	Ubiquinol oxidase 4, chloroplastic/chromoplastic
	isotig29943	GO:0007283 (spermatogenesis)	0.53	RNA pseudourine synthase 2, chloroplastic
	isotig31981	GO:0009793 (embryo development ending in seed dormancy)	2.08	Fatty acid amide hydrolase
	isotig70756	GO:0009910 (negative regulation of flower development)	0.59	Dolichol kinase
CK3 vs. TR3	isotig16582	GO:0048443 (stamen development)	1.37	Purple acid phosphatase 2
	isotig24901	GO:0003006 (developmental process involved in reproduction)	0.5	Serine carboxypeptidase-like 35
	isotig30606	GO:0010228 (vegetative to reproductive phase transition of meristem)	0.31	60S ribosomal protein L17-2
CK4 vs. TR4	isotig07103	GO:0009908 (flower development), GO:0010154 (fruit development)	1.72	Myb-related protein MYBAS1
	isotig08231	GO:0009553 (embryo sac development)	0.38	WAS protein family homolog 1
	isotig16158	GO:0048575 (short-day photoperiodism, flowering)	0.42	Ubiquinol oxidase 4, chloroplastic/chromoplastic
	isotig18119	GO:0010154 (fruit development), GO:0009911 (positive regulation of flower development)	0.41	Methyltransferase-like protein 16
	isotig30702	GO:0007291 (sperm individualization), GO:0007290 (spermatid nucleus elongation)	0.22	NA
	isotig44844	GO:0048510 (regulation of timing of transition from vegetative to reproductive phase)	1.19	1-aminocyclopropane-1-carboxylate oxidase
	isotig83604	GO:0009553 (embryo sac development), GO:0009908 (flower development), GO:0009909 (regulation of flower development)	0.65	NA

### MADS-box genes show high expression levels in male buds or female cone setting shoots, and play important roles in the transition from vegetative to inflorescence meristem identity

**Figure 6:**
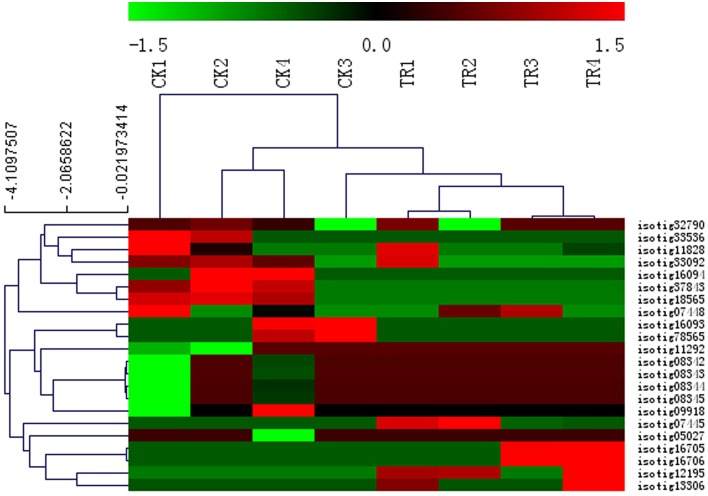
**Heat map showing the relative expression level for** ***Metasequoia*** **MADS-box genes in different stages during vegetative to reproductive phase transition based on DGE data analysis**. The color scale (−1.5 to 1.5) represents the Z-score calculated by comparing Reads Per Kilobase of exon model per Million mapped reads (RPKM). Hierarchical clustering of genes and samples was shown in the dendrogram on the top and side of the heatmap using the complete linkage approach.

Several studies on various plant species have shown that MADS-box genes control various aspects of development and reproductive processes including flower formation. Among the DE genes in our comparisons, genes involved in flower development were identified, including 22 *MADS*-box family genes with 10 putatively coding for *AGL MADS*-proteins, five *MADS*-box proteins *GGM*13, and seven *MADS*-box transcription factors. To examine the expression patterns of the 22 *MADS*-box candidate genes in all samples, we clustered the genes in a heat-map. We observed that the upregulated genes were found in the male and female buds compared to non-cone setting samples, such as isotig16705 and isotig16706 putatively coding for AGL19 (which are upregulated in female buds), and isotig07445 putatively coding for AGL24 (which is highly expressed in male buds) (Figure 6). This analysis provides expression conditions for proteins involved in the transition of vegetative growth to reproductive phase and floral induction. Phylogenetic relationship of MADS box genes from angiosperms and gymnosperms show homolog to the putative transcripts from *Metasequoia* PUTs (isotig33092, isotig32790, isotig16705, isotig16706, and isotig07445). These five genes belong to a subclass of a MADS box family called MIKC type (Supplementary Figure S2).

### Identification of DE genes related to hormone induction

A total of 34 genes were involved in hormone regulation (Supplementary data 4), including Auxin response factor 6, *ILR1*-like 1, IAA-amino acid hydrolase ILR1, ABC transporter B and G family member, Auxin efflux carrier component 1, and Protein TORNADO 2. Two homeotic transcripts (isotig10488 and 10489) putatively coding for Auxin-responsive protein IAA2 were also upregulated in TR (CK2 vs. TR2). During the female bud initiation stage, two homologous genes (isotig15995 and isotig15996), both putatively coding for IAA-amino acid hydrolase ILR1-like 1, showed increased expression levels (by 1.72-fold) in female cone setting shoots in CK3 vs. TR3 (Table 5).

**Table 5:** **DE genes related to hormone signals in the vegetative to reproductive phase transition**.

**Comparisons**	***Mg*PUTs ID**	**Differential Expression**	**Log_2_**-**fold**	**Annotation**
CK1 vs. TR1	isotig17616	Down	−0.42	ABC transporter B family member 21
	isotig35018	Up	1.29	ABC transporter G family member 35
CK2 vs. TR2	isotig23249	Down	−1.61	ABC transporter B family member 19
	isotig57304	Down	−3.13	ABC transporter G family member 6
	isotig10488	Down	−3.19	Auxin-responsive protein IAA2
	isotig10489	Up	1.03	Auxin-responsive protein IAA2
	isotig12557	Up	1.00	Auxin response factor 6
	isotig12558	Down	−0.84	Auxin response factor 6
	isotig13747	Down	−0.88	Auxin response factor 4
	isotig13748	Down	−0.58	Auxin response factor 4
	isotig14247	Down	−1.08	Auxin efflux carrier component 4
	isotig23227	Down	−1.12	Auxin response factor 7
	isotig25352	Down	−0.61	Auxin-responsive protein IAA13
	isotig44048	Down	−0.86	Auxin response factor 23
CK3 vs. TR3	isotig49246	Down	−1.80	ABC transporter G family member 34
	isotig14069	Down	−0.69	ABC transporter B family member 1
	isotig16667	Down	−0.60	Protein TORNADO 1
	isotig23272	Down	−0.52	Auxin response factor 6
	isotig23514	Down	−0.68	Auxin response factor 2
	isotig23745	Down	−0.38	Protein AUXIN SIGNALING F-BOX 3
	isotig26177	Down	−0.63	Auxin response factor 18
	isotig26345	Down	−0.36	Auxin response factor 4
	isotig46416	Down	−0.69	Auxin-responsive protein IAA30
	isotig15995	Up	1.73	IAA-amino acid hydrolase ILR1-like 1
	isotig15996	Up	1.74	IAA-amino acid hydrolase ILR1-like 1
	isotig43347	Up	1.99	IAA-amino acid hydrolase ILR1-like 6
CK4 vs. TR4	isotig04777	Up	0.55	IAA-amino acid hydrolase ILR1-like 1
	isotig04778	Up	0.56	IAA-amino acid hydrolase ILR1-like 1
	isotig28695	Up	0.36	Transcription factor ILR3
	isotig23576	UP	0.56	Auxin-responsive protein IAA26
	isotig07811	Up	0.99	ABC transporter B family member 21
	isotig25815	Up	0.43	ABC transporter B family member 20
	isotig30233	Up	0.57	ABC transporter B family member 2
	isotig52347	Up	2.23	ABC transporter B family member 4

### DE genes involved in starch and sucrose metabolism and the glycolysis/gluconeogenesis pathway

Annotated transcripts involved in important pathways were mapped to the KEGG (Ogata et al., 1999; Hashimoto et al., 2006) pathways using Blast2GO software (Conesa et al., 2005; Gotz et al., 2008). A total of 243 KEGG pathways (Supplementary data 5) were covered, among which 289 transcripts were found in the pathway of starch and sucrose metabolism. A total of 39 transcripts were significantly DE in TR compared to CK. One transcript was upregulated and 13 transcripts were downregulated in the TR for male bud initiation (CK1 vs. TR1 and CK2 vs. TR2). A total of 25 transcripts related to starch and sucrose metabolism were identified; among them, the transcript isotig37134, potentially coding for UDP-glycosyltransferase 85A1, was upregulated during the reproductive bud initiation stage (TR2 vs. TR3 and TR2 vs. TR4). In the glycolysis/gluconeogenesis pathway, 23 DE genes were identified in comparisons of TR with CK (CK1 vs. TR1, CK2 vs. TR2, CK3 vs. TR3, and CK4 vs. TR4). Among them, there was a common gene (isotig17002) putatively encoding bifunctional dihydroflavonol 4-reductase/flavanone 4-reductase in CK1 vs. TR1, CK2 vs. TR2, and CK3 vs. TR3. This gene showed upregulation in comparisons of CK1 vs. TR1 and CK3 vs. TR3, but downregulation in CK2 vs. TR2. This suggested that the glycolysis response for this gene was immediately induced after hormone treatment, but decreased after the transition phase (Supplementary data 6).

### Validation of illumina sequencing data by qRT-PCR

To validate the Illumina sequencing data, 32 DE genes identified by Illumina sequencing were tested by RT-PCR. The *Ct*-values of 18S rRNA of all samples ranged from 25.3 to 27.9 (Supplementary data 7). Selected genes included 17 upregulated genes and 15 downregulated genes. Among them, 29 genes (14 upregulated and 15 downregulated) were consistent between the two expression analysis platforms, with 92.3% showing the same trend of up- or down-regulation (Table 6). The correlation of expression measurements between these 29 consistent genes was 0.81 (*R*^2^ = 0.81) (**Figure 8**). Overall, the qRT-PCR and RNA-Seq analyses were consistent.

**Table 6:** **Selected DE genes tested with RT-PCR**.

**Comparison**	***Mg*PUTs ID**	**Log_2_ fold**	**RT-PCR 2^−ΔΔCT^**	**Regulation**	***p*-Value**	**Annotation (Blastx)**
CK1 vs. CK2	isotig37843	1.27	20.38 ± 5.08	Up	0.03	Agamous-like MADS-box protein AGL14 OS = *Arabidopsis thaliana*
	isotig24896	−1.02	0.92 ± 0.04	Down	0.05	Protein CCA1 OS = *Arabidopsis thaliana*
CK1 vs. CK3	isotig13090	2.09	4.8 ± 0.16	Up	0.02	Protein SUPPRESSOR OF npr1-1, CONSTITUTIVE 1 OS = *Arabidopsis thaliana*
	isotig43143	−2.05	0.71 ± 0.20	Down	0.03	Serine/arginine-rich splicing factor 33 OS = *Arabidopsis thaliana*
CK1 vs. CK4	isotig16184	2.8	4.58 ± 0.90	Up	0.01	Floricaula/Leafy-like protein FL1
	isotig07448	−1.81	0.83 ± 0.09	Down	0.04	MADS-box protein GGM13 OS = Gnetumgnemon
CK1 vs. TR1	isotig03483	2.56	3.47 ± 0.96	Up	0.03	Enzymatic polyprotein OS = Cauliflower mosaic virus (strain Strasbourg)
	isotig34815	−0.55	0.92 ± 0.06	Down	0.00	Zinc finger CCCH domain-containing protein 56 OS = *Oryza sativa* subsp. japonica
CK2 vs. CK3	isotig59775	1.45	4.24 ± 2.24	up	0.04	Transcriptional regulator STERILE APETALA OS = *Arabidopsis thaliana*
	isotig37873	−1.08	0.74 ± 0.16	Down	0.01	Zinc finger protein CONSTANS-LIKE 9 OS = *Arabidopsis thaliana*
CK2 vs. CK4	isotig38408	7.16	73.3 ± 16.9	Up	0.03	CASP-like protein 5 OS = Piceasitchensis
	isotig28528	−0.88	0.32 ± 0.08	Down	0.02	Floral homeotic protein APETALA 2 OS = *Arabidopsis thaliana*
CK2 vs. TR2	isotig76810	2.35	6.18 ± 1.26	Up	0.02	Putative receptor-like protein kinase At3g47110 OS = *Arabidopsis thaliana*
	isotig29034	−1.82	0.38 ± 0.11	Down	0.02	Galactinol synthase 1 OS = Ajugareptans
CK3 vs. CK4	isotig04971	2.07	33.82 ± 17.61	Up	0.02	Floral homeotic protein APETALA 2 OS = *Arabidopsis thaliana*
	isotig13666	−1.82	0.7 ± 0.27	down	0.02	Zinc finger protein CONSTANS-LIKE 10 OS = *Arabidopsis thaliana*
CK3 vs. TR3	isotig43347	1.99	0.25 ± 0.08	Up	0.01	IAA-amino acid hydrolase ILR1-like 6 OS = *Oryza sativa* subsp.
	isotig12601	−1.19	0.004 ± 0.002	Down	0.00	Zinc finger CCCH domain-containing protein 18 OS = *Arabidopsis thaliana*
CK4 vs. TR4	isotig52347	2.23	5.33 ± 2.68	Up	0.01	ABC transporter B family member 4 OS = *Arabidopsis thaliana*
	isotig04515	−4.7	0.02 ± 0.003	Down	0.02	Transposon Ty3-I Gag-Pol polyprotein OS = *Saccharomyces cerevisiae*
TR1 vs. TR2	isotig63887	4.26	18.03 ± 18.30	Up	0.00	Retrovirus-related Pol polyprotein from transposon TNT 1-94 OS = Nicotianatabacum
	isotig07445	−1.59	0.25 ± 0.03	Down	0.02	MADS-box protein AGL24 OS = *Arabidopsis thaliana*
TR1 vs. TR3	isotig27899	3.29	3.91 ± 0.60	Up	0.03	GDSL esterase/Lipase At2g42990 OS = *Arabidopsis thaliana* GN = At2g42990
	isotig14517	1.56	3.45 ± 1.28	Up	0.00	Floricaula/Leafy-like protein OS = Pinusradiata
TR1 vs. TR4	isotig59904	3.25	6.28 ± 1.98	Up	0.00	Zinc finger CCCH domain-containing protein 18 OS = *Arabidopsis thaliana* GN = At2g05160
	isotig11828	−2.18	0.62 ± 0.19	Down	0.04	Agamous-like MADS-box protein AGL15 OS = *Arabidopsis thaliana*
TR2 vs. TR3	isotig78463	2.64	0.02 ± 0.01	Up	0.04	Protein TsetseEP OS = Glossinamorsitansmorsitans
	isotig03422	−2.99	0.33 ± 0.06	Down	0.01	Retrotransposable element Tf2 155 kDa protein type 1 OS = *Schizosaccharomyces pombe*
TR2 vs. TR4	isotig59775	2.35	4.11 ± 0.89	Up	0.03	Transcriptional regulator STERILE APETALA OS = *Arabidopsis thaliana*
	isotig25237	−1.2	0.22 ± 0.01	Down	0.01	Zinc finger protein CONSTANS-LIKE 14 OS = *Arabidopsis thaliana*
TR3 vs. TR4	isotig43751	4.67	6.552 ± 0.758	Up	0.03	Transcription factor RAX3 OS = *Arabidopsis thaliana*
	isotig42187	−2.11	3.10 ± 0.10	Down	0.03	Zinc finger protein CONSTANS-LIKE 6 OS = *Arabidopsis thaliana*

### Levels of non-structural carbohydrates

In vegetative buds, fructose level did not fluctuate during May and June, with a decrease in July (Supplementary Figure S3). In contrast, the glucose level steadily increased during May to July. Similar to glucose, sucrose level increase in June followed by a decrease in July. During male bud initiation, the glucose level in male buds were three times higher than in vegetative buds 11 days after hormone treatment and then declined to the same level as vegetative buds. During female bud initiation, the glucose level in female buds were lower than in vegetative buds. Compared to vegetative buds, reproductive buds also had lower sucrose and fructose contents during May to July.

## Discussion

To expand our understanding of the gene expression network involved in the transition from vegetative to reproductive phases in *Metasequoia*, tag sequencing was used as a tool to mine genes (Marioni et al., 2008), because it performs better in gene discovery and has a higher dynamic range (Robinson et al., 2010). Based on our previous report of the first *Metasequoia* EST dataset, we developed these expression profiles using DGE Illumina's sequencing to analyze DE genes. Our results demonstrate that deep sequencing of nucleotide DGE tags can be used to resolve expression profiles and to detect differences in transcript abundance over a broad dynamic range. In our test case, which compared hormone-treated shoots to control shoots in four stages for male bud and female bud transition, we identified 69,520 (71.3%) transcripts in a previous database of 24 DGE libraries. Results from this expression analysis provide testable hypotheses to increase our understanding of the functions of DE genes involved in the vegetative-to-reproductive phase transition (male and female bud transition stage) via hormonal induction. In addition, glycolysis/gluconeogenesis was shown to play a role during floral development.

We identified a large number of putative transcripts that were differentially expressed only in comparisons of TR vs. CK. In the male bud initiation stage, some transcripts putatively coding MADS-box transcription factors were actively expressed during early flower development. For example, isotig32790, putative coding for MADS-box transcription factor 50 was one of the candidates, and was downregulated in TR3 compared to TR1, TR4 compared to TR1, and CK4 compared to CK2, but was upregulated in TR1 compared to CK1. These results suggest that this gene may act as a vegetative growth repressor and an early flowering inducer, especially as the male bud inducer. Another candidate gene that could have been annotated as being upregulated in male bud setting shoots is isotig33092, putative coding for *AGL*21, which shows a similar expression pattern to isotig32790, and is downregulated in CK4 compared to CK2, but is upregulated in TR1 compared to CK1 (Table 2). Several angiosperm genes in MADS gene family have been shown to act as floral integrators during the transition from vegetative-to-reproductive phase (Dorca-Fornell et al., 2011), but other genes such as *AGL*14 and *AGL*19 play significant roles in root development (Alvarez-Buylla et al., 2000). In gymnosperms, *DAL* 19, which is in the same clade as *AGL*42, is thought to have a similar function and to be involved in the transition from vegetative-to-reproductive growth (Uddenberg et al., 2013). Interestingly, in *Arabidopsis, AGL*21 (belonging to Type I MADS domains) is expressed in developing embryos and roots after germination (Burgeff et al., 2002), and is expressed in the male bud initiation before the phase change from vegetative-to-reproductive growth. These results suggest that the expansion of isotig33092 putatively coding for *AGL*21 contributed to transition from vegetative-to-reproductive growth, especially during male floral initiation.

The regulation of flowering time is a complex process with many environmental and internal signals, and a switch from vegetative-to-generative development at the appropriate time. During this process, MADS-box genes have been shown to play important roles in most aspects of angiosperm development, especially in fruit development (de Folter et al., 2005; Gramzow and Theissen, 2010; Lovisetto et al., 2012). In a recent report on the genome of the Norway spruce, it was shown that 278 MADS-box homologs are involved in phase changes in gymnosperms (Nystedt et al., 2013). A total of 22 PUTs with clear homology to MADS boxes were identified in our *Metasequoia* assembly (Figure 6). Isotig07445 putatively coding for the MADS-box protein *AGL*24 showed the highest expression during the early stage of male bud formation. Previous studies have revealed that this gene is a positive regulator of flowering in *Arabidopsis*. Overexpression of *AGL24* results in early flowering (de Folter et al., 2005). In the present study, isotig16705 and isotig16706 putatively coding for *AGL19* was highly expressed in the female bud formation stage (TR3 and TR4). Previous reports have shown that this gene is involved in root development. Thus, it is important to identity these genes in gymnosperms. We propose that isotig07445, isotig16705, and isotig16706 may play an important role in reproductive bud formation during the transition phase of *Metasequoia*. However, further analyses about the transcript function are required to increase our understanding of the transition phase from vegetative to reproductive phases in gymnosperms.

Recent studies suggest that both *FCA* and *FPA* function in the independent (autonomous) pathway to control floral inhibitor FLOWERING LOCUS C (*FLC*) mRNA accumulation and flowering time (Schomburg et al., 2001; Feng et al., 2011). In our expression profiles, we did not identify *Metasequoia FLC* homologs or they did not show any DE. The *Arabidopsis* gene *FCA* encodes an RNA-binding protein that promotes floral transition and root development (Macknight et al., 2002). Interestingly, previous studies have shown that *FCA* is an ABA receptor (Hirayama and Shinozaki, 2007). In our experiments, isotig14235 and isotig14236 putatively coding *FCA* showed increased expression in hormone-treated shoots during female bud formation, while was downregulated during vegetative development (Table 3). Therefore, our data suggest that *FCA* may be activated in the female bud transition stage and is involved in the female bud formation development in gymnosperms. In *Arabidopsis, FPA* has been identified as a regulator of flower development and enables flowering by limiting expression of the floral repressor *FLC* (Michaels and Amasino, 2001; Schomburg et al., 2001). However, the biological role of *FPA* is not restricted to flowering, but also influences other aspects of development (Veley and Michaels, 2008). Our data suggest that isotig12610 and isotig12611 putatively encoding *FPA* in *Metasequoia* repress male bud initiation, but promote vegetative bud initiation. *LEUNIG* is a key regulator to repress the *Arabidopsis* floral homeotic gene *AGAMOUS*, which regulates the activity of both floral meristem identity genes as well as floral organ identity genes (Conner and Liu, 2000). *LEUNIG_HOMOLOG* and *LEUNIG* also play a role during *Arabidopsis* embryo and floral development by forming a putative corepressor complex with another protein, SEUSS (*SEU*) (Sitaraman et al., 2008). However, during the female transition stage, upregulation of isotig78340 putatively coding *LUG* in hormone-treated shoots compared to the control may suggest that it is a floral transition inducer. However, further studies are required to characterize the gymnosperm-specific genes involved in the female transition phase. Recent studies have shown that zinc finger CCCH domain-containing proteins regulate flowering time and play significant roles in developing floral tissue (Colasanti et al., 2006; Chao et al., 2014). In addition, the CONSTANS family of transcription factors, some of which play important roles in photoperiod-induced floral induction, have a unique combination of zinc fingers. For example, zinc finger protein CONSTANS-LIKE genes play a significant role in regulating flowering in *Arabidopsis* and rice (Griffiths et al., 2003; Wenkel et al., 2006; Tsuji et al., 2011). In our TR2 vs. TR3, TR1 vs. TR2, and TR1 vs. TR3 comparisons, all of the transcripts putatively coding the zinc finger CCCH domain-containing proteins were upregulated in the samples collected at earlier time points, while in TR3 vs. TR4, most of the zinc finger CCCH were also upregulated, suggesting that these transcripts may play a positive role in bud initiation.

**Figure 7:**
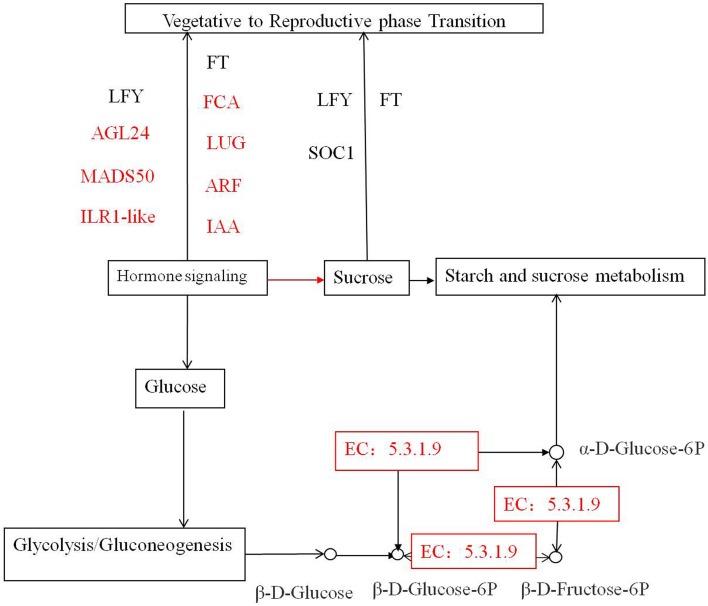
**Multiple interactions among the genes involved in vegetative to reproductive phase transition and pathways of starch and sucrose metabolism and glycolysis/gluconeogenesis in response to hormone signaling**. Colored rectangles correspond with the *Matesequoia* genes detected using DGE.

In the starch and sucrose metabolism pathways (Figure 7, Supplementary data 6), we observed upregulation of isotig37134 putatively coding for UDP-glycosyltransferase 85A1 in TR2 vs. TR3 and TR2 vs. TR4, and upregulation of the transcripts putatively coding for ADP, ATP carrier protein in TR2 vs. TR4, indicating that these key transcripts are required for bud formation and flowering development. Glycosyltransferases (GTs) are enzymes involved in the modification of secondary metabolites, phytohormones, and xenobiotics (Kumar et al., 2011) that transport sugar molecules to the specific acceptors and form glycosidic bonds. The glycosylation reaction is often the final step in plant natural product synthesis, which stabilizes and provides a safe storage form for potentially toxic aglycones. The UGTs transfer the glycosyl group from an activated nucleoside diphosphate sugar donor (UDP-sugar) to a wide range of sugar or non-sugar acceptors (Sharma et al., 2014). Recently, the UDP-glycosyltransferase 85B1 model in Sorghum has been used to identify cations of amino acid residues important for the catalysis and binding of sugar donors and acceptors in the active site (Thorsoe et al., 2005). However, it remains unclear how the sugar donor specificity of UDP-glycosyltransferase 85A1 has changed. Identification of *Metasequoia* transcripts putatively coding UGTs can be used to develop plants that react efficiently to stress conditions and may be used for functional analysis. The 20 downregulated genes that potentially play a role in pectinesterase activity (GO:0030599) in TR3 vs. TR4, CK2 vs. TR2, CK3 vs. TR3, and TR2 vs. TR4 comparisons may block carbon flow to pectate (Zhang et al., 2014). During glycolysis/gluconeogenesis, downregulation of transcripts putatively coding alcohol dehydrogenase (NAD) and glutathione dehydrogenase in CK2 vs. TR2 may slow the entry of carbon into the citrate cycle, pyruvate metabolism, and propanoate metabolism, leading to less α-D-glucose-6P being converted into glycerate-3P and α-Dglucose-1P. As a result, the majority of α-D-glucose-6P is transported into the amyloplast for starch and sucrose metabolism. These results suggest that these enzymes are rate-limiting in two important pathways during vegetative bud formation. These findings may facilitate the breeding of *Metasequoia* to enhance starch accumulation during vegetative to reproductive bud transition and increase our understanding of biological processes in bud formation.

In Figure 7, we show that the effects of the sugar-hormone interplay may be mediated by hormones that enable tissues to respond to sugars, resulting in vegetative-to-reproductive phase transition. At the same time, hormone and sugar signaling may independently affect vegetative-to-reproductive phase transition (Cho and Yoo, 2011; Matsoukas, 2014). The actions of all pathways finally converge to control the expression of some floral pathway integrators. In our expression tags, genes putatively coding for auxin response protein (IAA) and ARFs respond to hormone regulation during male and female bud transition. In a previous study of endogenous hormones levels during initiation stage (early June to early August) (Liang and Yin, 1994), we found that while *Metasequoia* reproductive buds shared similar patterns of IAA, ABA, and GA_1+3_ dynamics with vegetative buds, the former showing higher hormone contents and greater fluctuations with female buds having the highest contents of the analyzed hormones followed by male buds. The current study substantiated our previous findings with the identified DE transcripts involved in hormone synthesis pathways. For instance, most, if not all, of the DE transcripts with a GO function of gibberellin, IAA, or ABA biosynthetic process, were upregulated in reproductive buds when compared to vegetative buds of the same stage. pathway integrators including transcripts putatively coding AGL24, MADS50, FCA, and LUG were found to regulate male and female phase transition, and flowering locus T (MFT), SOC1, and LEAFY (LFY) identified in our previous report play roles in the late stages (Lee et al., 2008; Zhao et al., 2013). Here, we highlight the potential roles of sugar-hormone interactions in regulating the vegetative to reproductive phase transition; however, further studies are required to understand the antagonistic and agonistic interactions between the sugar and hormone signals in a spatiotemporal manner at the molecular level. Their association with other transcriptional regulatory networks remains unclear, so functional studies of these genes in *Metasequoia* remain to be done.

Biochemical or physiological changes, such as sugar, can be recognized during the transition of a vegetative apex into a floral apex. The non-structural carbohydrates tested in the present study (sucrose, glucose, and fructose) had a lower level in reproductive buds than in vegetative buds in *Metasequoia*, with the exception in late May and mid June for glucose. This is consistent with the finding of a decrease of sucrose level in strawberry shoot tips at the beginning of floral induction (Eshghi and Tafazol, 2006). We also found that the glucose level during early male bud initiation was thrice higher than in vegetative buds. These observations are supported by the differential expression of related genes. Isotig26509 involved in gluconeogenesis was found induced in comparisons of CK1 vs. TR1, CK1 vs. CK3, and CK1 vs. CK4. Isotig18149 with an annotation of galactinol-sucrose galactosyltransferase activity was also upregulated in female buds when compared to vegetative buds. Collectively, our data suggest that soluble carbohydrate contents may play a role in *Metasequoia* floral induction: lower levels of sucrose and fructose facilitate initiation of both types of reproductive buds, while elevated glucose content is favorable in male bud initiation and the opposite in female buds.

In a previous study, we identified 17,086 PUTs in late female bud initiation stage that were not found in vegetative buds during late August and early September (Zhao et al., 2013). In early spring (March) when buds rapidly enlarged, 25,643 PUTs were found uniquely in female cones. Our current study identified a total of 6166 DE PUTs between reproductive and vegetative bud initiation (late May–early July), which was the most DE PUTs among all comparisons. Clearly the hormone treatments induced significant transcriptomic changes that led to the vegetative apex into a floral apex. It is worth noting isotig63639, a DE PUT putatively encoding a histone deacetylase. This type of the enzymes allows histone proteins to wrap the DNA more tightly leading to low or no expression of target genes. Isotig63639 was found downregulated in TR1, suggesting that target genes were turned on or expressed at a higher level upon hormone treatments. In addition, while both male and female buds showed decreased expression of *ELF3*, Flowering time control protein FPA and Zinc finger CCCH domain-containing protein encoding genes, differences in existed between male and female buds during their initiation (Tables 2, 3). Transcript isotig32790 putatively coding for MADS-box transcription factor 50 showed upregulation in male bud initiation and downregulation in female bud initiation. Another upregulated transcript in male bud initiation encoded an Agamous-like MADS-box protein AGL21. In contrast, PUTs for transcriptional corepressor LEUNIG and Flowering time control protein FCA were found upregualted in female bud initiation. There results were consistent with the differences of male and female buds in initiation time, hormone response, and sugar contents.

**Figure 8:**
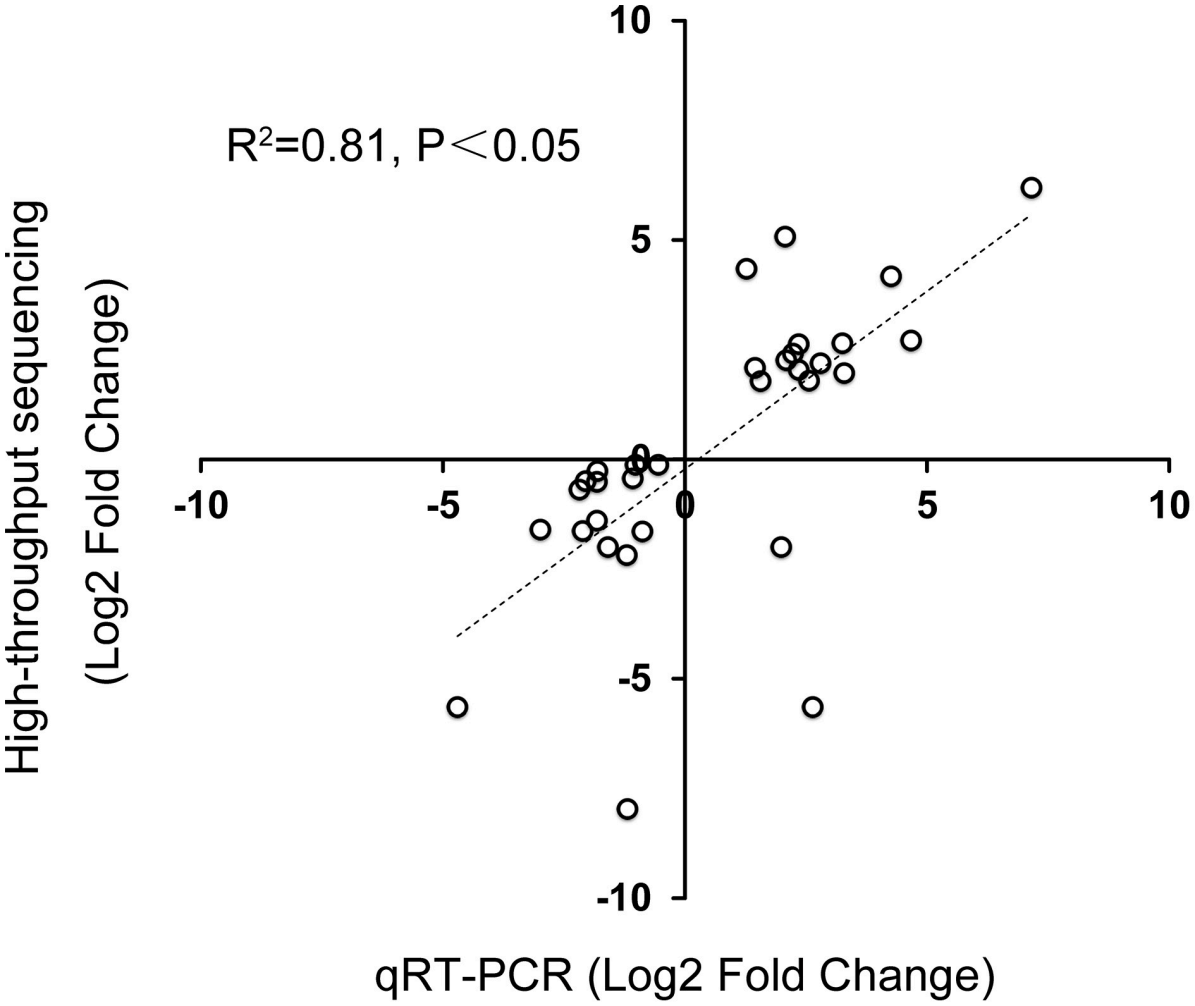
**Correlation between qPCR and Illumina sequencing data**. Correlation between qRT-PCR and Illumina sequencing data of 32 selected genes: 17 upregulated genes and 15 downregulated genes in 16 pairs of amplified RNA samples. Spearman Rank Correlation coefficient = 0.81 (*P* < 0.05).

In summary, the present study identified numerous DE genes among the four stages of bud development, and provided novel information on female and male bud initiation. The results from the *in silico* DE analyses were reliable because 29 out of the 32 genes chosen for qPCR showed the same upregulation or downregulation trend as the *in silico* analyses (Table 6, Figure 8). Overall, this study identified a large number of DE genes regulated in the male and female bud transition and provides new insights into the interaction and overlapping genes in the sugar-mediated flowering pathway during the bud-formation stage.

### Conflict of interest statement

The authors declare that the research was conducted in the absence of any commercial or financial relationships that could be construed as a potential conflict of interest.
