# Supplementary material for: Digital gene expression analysis of male and female bud transition in Metasequoia reveals high activity of MADS-box transcription factors and hormone-mediated sugar pathways
Source: Front Plant Sci. 2015 Jun 24;6:467. doi: 10.3389/fpls.2015.00467 (PMC4478380; doi:10.3389/fpls.2015.00467)

Supplementary Fig.S1: A: Longitudinal sections of male bud, the arrows show pollen; B: Longitudinal sections of female bud, the arrows show female bud meristem. Scale bars=200μm

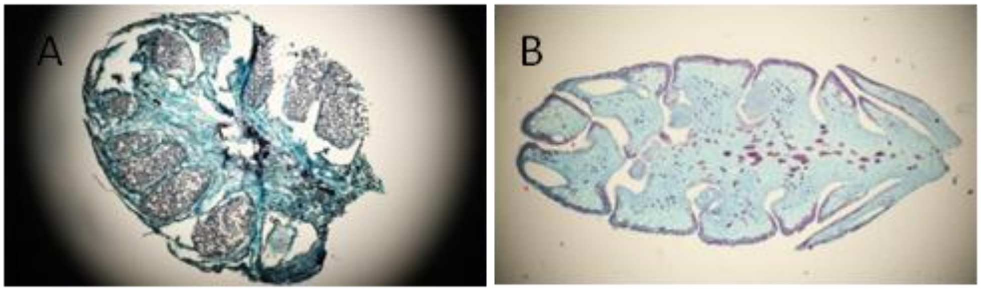

Supplementary Fig.S2: Phylogenetic relationship of MADS box,genes from angiosperms and gymnosperms that show homolog to the putative transcripts (isotig33092, isotig32790, isotig16705, isotig16706, isotig07445).

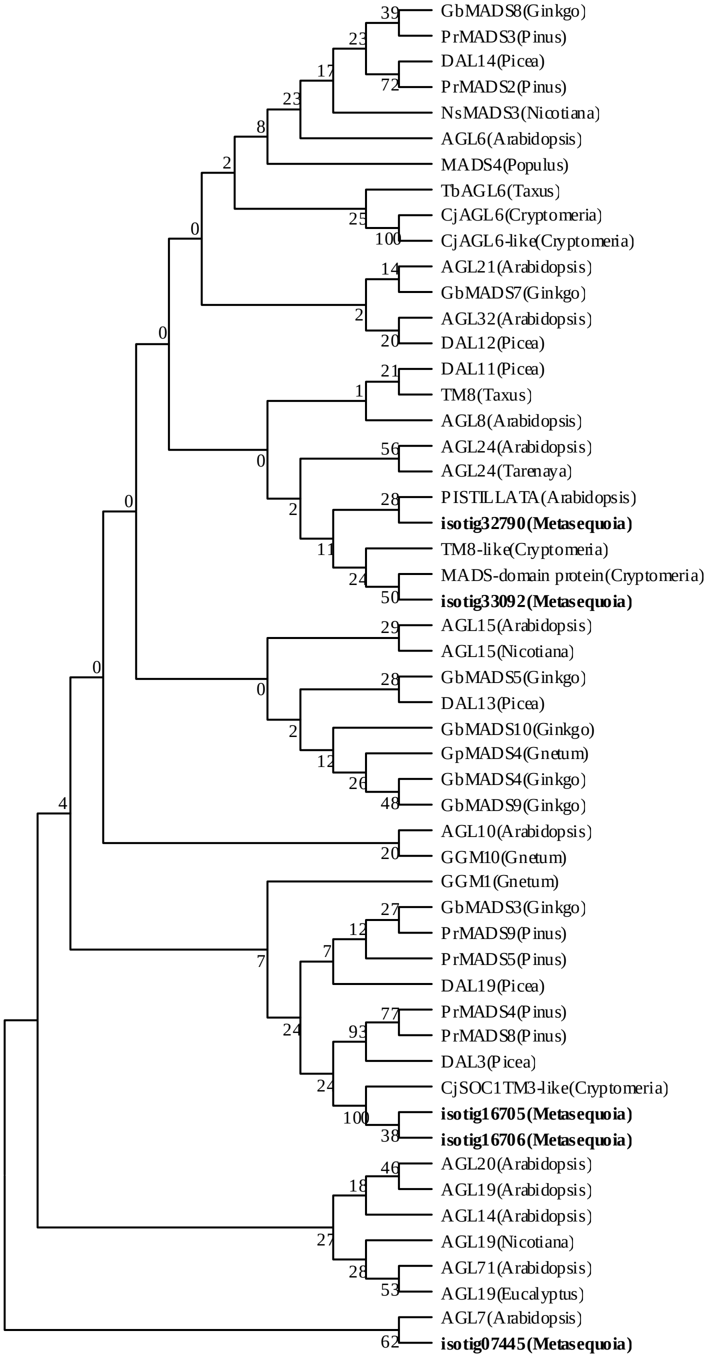

Supplementary Fig.S3: Sugar measurement. A: Glucose content meaturement; B: Sucose meaturement; C: Fructose meaturement. Samples of female, male and vegetative buds on stems used for DGE library were collected. The glucose, and sucrose and fructose, content was analyzed by GC-MS and quantified. Error bars indicate SD. The values with different letters on the bar were significantly different (P<0.01).

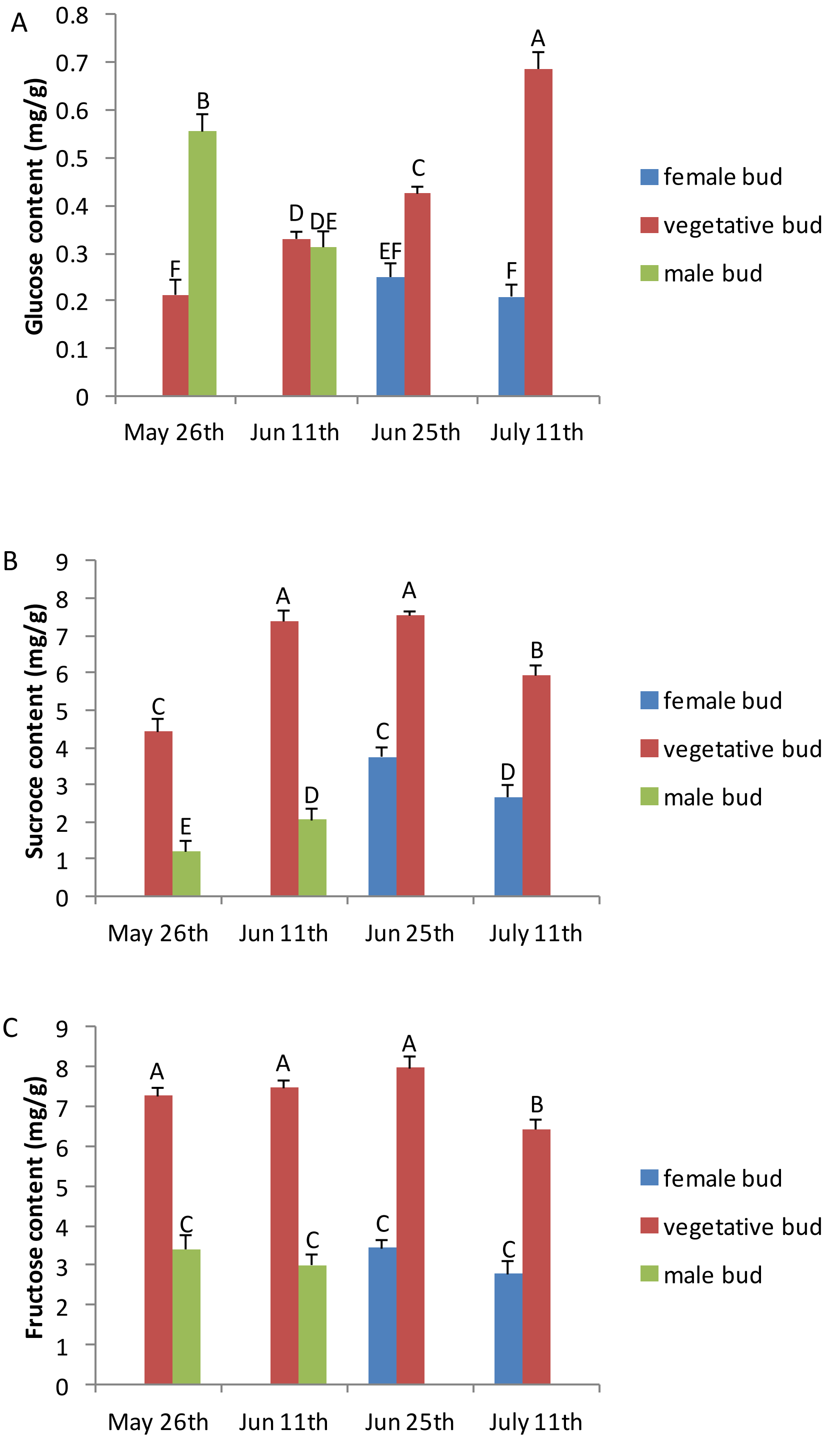

Supplement: Supplementary file 2 [file Image1.PDF]
